# Unravelling COVID-19 vaccination attributes worldwide: an extensive review regarding uptake, hesitancy, and future implication

**DOI:** 10.1097/MS9.0000000000000921

**Published:** 2023-06-12

**Authors:** Hossam T. Ali, Yara Ashour, Mohammed A. Rais, Mostafa Barakat, Tasnim A. Rezeq, Mohamed M. Sharkawy, Mina Lapic, Ziad A. Soliman, Ahmed Abdullah, Abdulrahman Allahham, Abdelaziz Abdelaal, Samar A. Amer, Ranjana Rohilla, Aroop Mohanty, Ranjit Sah

**Affiliations:** a Qena Faculty of Medicine, South Valley University, Qena; b Department of Public Health and Community Medicine, Family Medicine, Ministry Of Health (MOH) Saudi Arabia , Zagazig University; c Anesthesia and ICU Fellowship, Egyptian Ministry of Health; d Founder, Tanta Research Team; e Faculty of Medicine, Tanta University, El-Gharbia, Egypt; f College of Medicine, Sulaiman Alrajhi University, Albukayriah, Al-Qassim, Saudi Arabia; g Clinical Research Scholar; h MMSCI Candidate, Harvard Medical School, Boston; i Research Volunteer, Doheny Eye Institute, UCLA, USA; j Al-Quds University; k Ministry of Health, Gaza, Palestine; l Faculty of Medicine of Algiers, University of Algiers1, Algeria; m Membership at Royal College of General Practice [INT], London, United Kingdom; n Department of Clinical Microbiology, SGRRIMS, Dehradun, Uttarakhand; o Department of Clinical Microbiology, All India Institute of Medical Sciences, Gorakhpur, Uttar Pradesh; p Department of Public Health Dentistry, Dr D.Y. Patil Dental College and Hospital; q Department of Clinical Microbiology, DY Patil Medical College, Hospital and Research Center, DY Patil Vidyapeeth, Pune, Maharashtra, India; r Tribhuvan University Teaching Hospital, Kathmandu, Nepal

**Keywords:** coronavirus, COVID, hesitancy, uptake, vaccine

## Abstract

Since the declaration of the coronavirus disease 2019 pandemic, all efforts were directed towards limiting the transfer of the disease and preventing severe disease forms from occurring. In this regard, numerous vaccines were quickly developed to limit the associated morbidity and mortality of the disease and to reduce the burden on healthcare systems worldwide. However, to date, vaccine hesitancy remains a major limitation to vaccine distribution, with varying degrees in different countries. Therefore, the authors conducted this literature review to highlight the magnitude of this issue throughout the globe and summarize some of its major causes (i.e. governmental, healthcare system-related, population-related, and vaccine-related) and contributing factors (i.e. knowledge/awareness, social media, etc.). In addition, the authors highlighted some of the main motivating factors that can minimize the burden of vaccine hesitancy at the population, governmental, and worldwide levels. These include structural (i.e. government, country), extrinsic (i.e. family, friends), intrinsic (i.e. self-perception), and other factors (financial and nonfinancial). Finally, the authors proposed some implications for future research to ease the vaccination process and hopefully, put an end to this problem.

## Introduction

Highlights

- The declaration of the coronavirus disease 2019 pandemic, all efforts were directed towards limiting the transfer of the disease and preventing severe disease forms from occurring.
- However, to date, vaccine hesitancy remains a major limitation to vaccine distribution, with varying degrees in different countries.
- In addition, we highlighted some of the main motivating factors that can minimize the burden of vaccine hesitancy at the population, governmental, and worldwide levels.
- These include structural (i.e. government, country), extrinsic (i.e. family, friends), intrinsic (i.e. self-perception), and other factors (financial and nonfinancial). Finally, we proposed some implications for future research to ease the vaccination process and hopefully, put an end to this problem.

Throughout history, some emerging infectious diseases have imposed a real challenge on healthcare systems and the general population. For instance, the plague during WWII and influenza in the past^1^. In the last century, viral diseases have become the main challenging threat^2^.

The new severe acute respiratory syndrome coronavirus 2 (SARS-CoV-2) that causes respiratory disease with atypical presentation emerged in 2019. The first case was diagnosed in Wuhan, China, and shortly after that, it spread all over the world. The WHO declared the coronavirus disease 2019 (COVID-19) pandemic in March 2020^3^. As a respiratory disease with droplet transmission, it has spread vigorously throughout the world, imposing serious threats on the general population and the global healthcare system^4^.

According to the WHO, until 25 November 2022, there have been 636 440 663 confirmed cases of COVID-19, including 6 606 624 deaths^5^. Healthcare systems and governments had to develop and implement many preventive measures, the most important of which were vaccines. Manufacturers started to develop many vaccines within a short time. However, the process of vaccination was not accepted by the population. Some obstacles hindered this process, either within healthcare systems or through external factors^6^. These consequences have caused some countries to postpone vaccination. Thus, factors affecting vaccination uptake need to be considered and thoroughly investigated to propose measures that can ease the vaccination process and subsequently limit the burden on healthcare systems. Therefore, we conducted this literature review to highlight the magnitude of this issue throughout the globe and summarize some of its major causes (i.e. governmental, healthcare system-related, population-related, and vaccine-related) and contributing factors (i.e. knowledge/awareness, social media, etc.).

## Coronavirus illness-19 (COVID-19)

The coronavirus virus, which causes coronavirus illness, is a broad family of viruses and an enveloped positive-sense single-stranded RNA virus. It has become a major health issue in the last two decades. Severe acute respiratory syndrome coronavirus 1 occurred in 2003. Besides, sporadic cases of Middle East respiratory syndrome occurred in 2012 in certain geographic areas. These two serious events have alerted the world to the potential diseases that can occur in the 21st century^7^ including the severe acute respiratory illness, which is also known as coronavirus 2, or SARS-CoV-2, which was discovered to be a human pathogen in 2019. Both people and some animals can contract these viruses.

The virus is transmitted from person to person through droplets expelled during coughing, sneezing, or talking by an infected person. A less prevalent method of transmission is through contacting one’s mouth, nose, or eyes after touching a surface that has the virus on it. The droplets expelled during face-to-face contact constitute the most common mode of transmission. Another possible mode is contact surface spread. Transmission via aerosols has been controversial and unclear^4^.

The clinical presentation of COVID-19 cases can be widely variable, as the virus causes inflammatory reactions and affects many organs. from asymptomatic cases to severe cases that need hospitalization and may lead to death. The most common symptoms include fever, dry cough, fatigue, expectoration, sore throat, difficulty breathing, chills, nasal congestion, hemoptysis, anosmia, rhinorrhea, nausea, vomiting, diarrhea, headache, and confusion. The symptoms are not only confined to the respiratory system. Affection of organs such as the heart and kidney was not uncommon as a result of inflammatory and cytokine storms. Despite prophylactic treatment, some COVID-19 patients experienced a stroke. Acute coronary syndrome with normal coronaries, arrhythmias, and cardiomyopathy was reported in some COVID-19 cases. Acute kidney injury occurs especially in people with impaired baseline renal function^8^.

The COVID-19 epidemic has been managed and overcome through the WHO continuous efforts and initiatives as the disease spread around the world. There are no specific antivirals and immune therapies, and only a few have shown promise in lowering COVID-19 patients’ mortality rates. Treatment for COVID-19 and SARS-CoV-2 infection are the subjects of research. Therefore, it has been demonstrated that human adherence to international preventive measures, such as face masks, social withdrawal, prolonged quarantine, and travel restrictions, only goes so far in slowing the spread of the virus. Despite the efforts, morbidity and mortality rates did not decrease significantly. The creation of an efficient and widely available vaccine is the greatest method to contain and ultimately eradicate this epidemic. Vaccination was considered the most important and effective method to decrease the burden of the disease, the need for hospitalization, and the risk of developing complications^9,10^.

## COVID-19 vaccination

One of the 20th century’s greatest achievements in public health was vaccination, as more than 12 vaccines had been developed and authorized worldwide within only a year of the WHO’s declaration of COVID-19 as a pandemic. Following that, an increasing number of vaccines were developed^10^. With the long-term objective of developing herd immunity, high vaccination coverage is advocated as the primary public health strategy to manage and flatten the COVID-19 epidemic ‘curve’. However, in most cases, vaccine development can take years^11^.

According to the WHO, on 23 November 2022, there were 175 COVID vaccines in clinical development and 199 vaccines in preclinical development^6^. A total of 42 vaccines have already been approved for use. The most influential vaccine platforms are mRNAs such as those from Pfizer-BioNtech and Moderna, vaccines containing nonreplicating viral vectors such as those from Johnson & Johnson and Oxford/AstraZeneca, and inactivated virus vaccines such as those from Sinopharm. However, attention has shifted to protein subunit vaccines^12^. This huge progress in the vaccine market has increased the availability of vaccines in addition to their ease of use. Nasal vaccines and single dose vaccines are already in use, while oral vaccines are being developed^12^.

Even though many different types of vaccines are available, there is still a portion of the population that refuses to take the vaccine^13^. Indeed, this may have prolonged the pandemic in some countries, such as the USA^14^. Understanding and addressing hesitancy and antivaccine attitudes is thus critical^10^. Moreover, this pandemic has highlighted the vulnerabilities and weaknesses in healthcare systems all over the world^15^. Thus, it is essential to make good use of all of that to help prepare our hospitals and healthcare providers (HCPs) for any coming challenges.

## COVID-19 vaccination uptake

Although the vaccines and other protective measures have proven their effectiveness against the pandemic, people still need to have the desire to take the vaccine. Because of this, the public’s acceptability of the new COVID-19 vaccination is still unsure, despite its availability. One of the most challenging health issues is vaccine hesitancy, which the WHO views as a danger to world health^16^.

Some studies suggest that one in every five people reports hesitancy to get vaccinated^13^. In a study across 19 countries in 2020, 71.5% of participants responded that they would take a vaccine if it were proven safe and effective, this percentage increased to 75.2% in June 2021 among 23 countries^17,18^. The effort to protect family members was the most frequently chosen motive for vaccination while being exempt from antiepidemic measures after vaccination was the least frequently chosen motive^19^.

**Figure 1:**
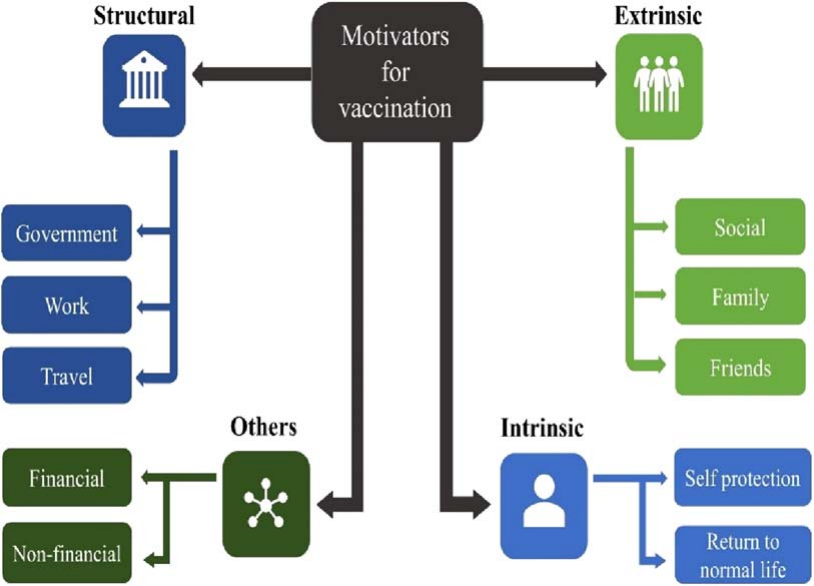
The determinants of COVID-19 vaccine uptake.

As illustrated in (Fig. 1); the determinants of uptaking the COVID-19 vaccine are extrinsic, intrinsic, and structural. Extrinsic motivators were reported as the single largest category of vaccine motivators among hesitant adopters. Extrinsic motivators are those affecting people’s decisions from a social and cultural point of view. According to studies, the broad prosocial motivation is to protect other people and families, help the community, and play their part in eradicating the virus. On the other side, a lot of individuals reported that the most common intrinsic motivator was to protect themselves from COVID. Other motivations or incentives include structural motivations or governmental mandates, which are suitable for hesitant people. Those people need to get vaccinated to get a government issue done. The most prominent structural motivation was related to travel, followed by work^20^.

The vaccination process has been improved using different approaches such as information provision and persuasion, financial incentives, and nonfinancial incentives (e.g. vaccine passes, travel restrictions, etc.).

### Intrinsic factors

#### The level of knowledge of the population about COVID-19 vaccines

During the era of COVID, a lot of wrong information was spread among the population. Easily accessible social media platforms have allowed the spread of medical misinformation and unverifiable content about the global COVID-19 epidemic^21^. Besides, low digital health literacy affects large percentages of populations around the world. It directly contributes to the spread of COVID-19-related online misinformation and its devastating effects. Moreover, the lack of clear evidence and expert opinion on those platforms has given the advantage to nonexpert opinions^22^.

In addition to that, the total knowledge score was significantly positively correlated with higher practice scores, and vice versa^23^. A cross-sectional survey of 1249 participants revealed that the extent of information regarding the COVID-19 vaccine and vaccination program was low, as approximately half of the participants did not know that children, adolescents, pregnant, and lactating women were eligible for the vaccine. Another important point is that information obtained from reliable sources such as government agencies and healthcare workers was significantly associated with socioeconomic status. The higher the socioeconomic status, the greater the influence from reliable sources. However, there was a lack of information about who was eligible to be vaccinated^24^.

A survey of the general population in Ghana in 2021 revealed that about 49% of participants did not want to take the vaccine or had not decided yet. The most common reasons for that were a lack of sure information about the vaccine’s safety, possible effects, and efficacy to prevent COVID-19 infection. Media and social media were the main sources of information among them. Another study of the Hong Kong population, which had early access to vaccines but a low vaccination rate, showed that hesitancy was associated with the safety and efficacy of certain vaccines supplied other than infection risk^25^. A recent study in China revealed that about 45% of people were hesitant about vaccination.

#### Self-protection

Protection Motivation Theory states that elements like vaccination views, efficacy, the severity of health threats, and a low incidence of community illnesses can affect a person’s motivation to get vaccinated, making them crucial components of engaging in healthy behavior. Particularly, worries about the risks or side effects, as well as social and peer pressure, can significantly affect a person’s willingness to get vaccinated^26,27^.

### External influences on the vaccination process

#### Social media

During the COVID-19 pandemic, the world became increasingly physically distant, with lockdown measures such as social distancing, disbanding of public gatherings, and remote work environments instituted in many countries worldwide. Consequently, social media grew to fulfill a critical role as a source of social news and the primary information outlet for governments and health organizations^28^. Different social media facilities make it easy for individuals to find health information. However, antivaccine groups were active on social media and spread misinformation, which also influenced willingness to vaccinate^29^. In February 2020, the WHO warned of an infodemic, a wave of fake news, and misinformation on social media concerning COVID-19. Additionally, after the approval of COVID-19 vaccines, misinformation about the vaccinations also started to disseminate quickly^30^. It was found that rumors and misinformation may lead individuals to generate and develop false beliefs about the effectiveness of vaccines. Thus, it has a significant negative effect on willingness to accept COVID-19 vaccines^28,30^.

Moreover, it was found that even a few minutes of exposure to antivaccine information on the internet can have a sharp impact on people’s attitudes toward vaccination risks^31^. Besides its detrimental effect on public health efforts, especially during a public health emergency, social media is considered a double-edged sword because it becomes a source of data for detecting outbreaks and understanding public attitudes and behaviors during public health emergencies^32^. Moreover, social media offers direct communication between HCPs and patients, is known to reduce vaccine concerns and improve overall uptake and has made health able to produce news content and public service announcements. This content should be designed to effectively educate and reassure broad, diverse audiences regarding key vaccine-related topics. Ideally, these media engagement efforts to build public trust in vaccines should be formulated and implemented as soon as possible^33,34^.

### Politics and the vaccination hesitancy

Nowadays, politics plays a key role in almost everything in the world. Thus, it has affected the process of COVID-19 vaccination. The acceptance or rejection of vaccines produced abroad is closely tied to the foreign policy preferences of sitting governments. In Brazil, President Jair Bolsonaro has been a firm critic of China since his inauguration in January 2019. That opposition has led him to openly criticize the vaccine developed by Sinovac Biotech, leading millions of Brazilians to reject it^35^.

According to the Council for Medical Schemes studies, not trusting the government’s ability to ensure that the vaccine is safe and effective, as well as believing that politics played an excessive role in the vaccine development process, accounted for 14 and 8% of the total reasons for not wanting to get the COVID-19 vaccination, respectively^36^. Governments that deliberately release reassuring misinformation about COVID-19 may also reduce its acceptance^37^. For example, a renowned political analyst in Pakistan claimed that the COVID-19 vaccine has nano-chips to control human bodies through the 5G internet. The ex-foreign ministry of Pakistan also made similar disinforming comments, accusing the United States of inventing the coronavirus in UK labs and then transferring it to China for spread^38^. As a result, the debate over COVID-19 vaccination has not been without political squabbles, undermining hopes of eradicating the virus.

### Governmental issues during the vaccination process

Governments usually have some issues during serious and catastrophic events. One of the governmental tools is the immunization mandate, which aims to allow the community to restore normal social and economic activities. Usually, these mandates are recommended in situations and activities in which social distancing is difficult or even impossible, like restaurants, public housing, nightclubs, and large sporting events^33,39^. Vaccine mandates for adults by state governments were considered acceptable by 40.9% of the US population, whereas 47.7% accepted mandates by their employers to attend work. However, a 2020 global study showed that people were potentially more likely to accept voluntary vaccination over employer-mandated vaccination^17^.

Although this tool can be effective for all population groups, school-aged children’s mandates should be considered alongside a high level of experience with vaccine safety and good education for the parents to prevent the consequences of keeping the children home. Thus, it would be more applicable and effective, especially for this group of the population^33^. In the United Kingdom, a study was conducted to assess the change in vaccination intention from baseline intent following the introduction of the vaccination passport. The results revealed that passports had a polarizing effect on the study population, which means that people who were provaccination had a positive opinion while those who did not want to vaccinate or had doubts had anger and negative emotions^39^. Anger and negative emotions are more likely to motivate actions to restore limited freedom (i.e. psychological reaction theory)^40^. So, vaccination mandates are less likely to be effective in increasing vaccination acceptance but can be used as a collective tool to increase the rate of vaccination^39,41^.

Government mistrust was associated with vaccine hesitancy in most countries. Respondents who said that they trusted their government were more likely to accept a vaccine than those who said that they did not. Yet, after accounting for sociodemographic and COVID-19 experience variables, Lazarus *et al.* found that mistrust in the central government was not significantly associated with vaccine hesitancy, as was also the case for the local government, in most countries sampled, except in Ghana and Poland. It was concluded that the association between trust in the government and vaccine hesitancy was not significant^17,18^.

### Financial and economic factors

Financial incentives (or subsidies) promote vaccine take-up by reducing the cost of vaccination^42^. Although vaccines are free in most countries, people bear indirect costs, which include costs related to transportation and foregone income, as well as psychological and monetary costs that may be caused by the side effects of vaccination. In India, most people (69.6%) were vaccinated in the private sector and can afford it. About 26.9% of people can partially afford the vaccine cost, and about 3% of people cannot afford the vaccine cost^43^. There is also a link between rewards and increased vaccination rates, as demonstrated in the United States. Offering about $100 resulted in a 2% increase in vaccination rates while offering about $500 resulted in a 50% increase in vaccination rates^44^. In Sweden, the modest financial incentives (US$24/SEK 200) increased the COVID-19 vaccine uptake rate by 4.2% from a baseline rate of 71.6%^45^. Paying individuals to get vaccinated can be justified, as vaccination against COVID-19 benefits the individual receiving the vaccine and their community, as broad vaccination decreases the number of hosts for the virus^44^.

**Figure 2:**
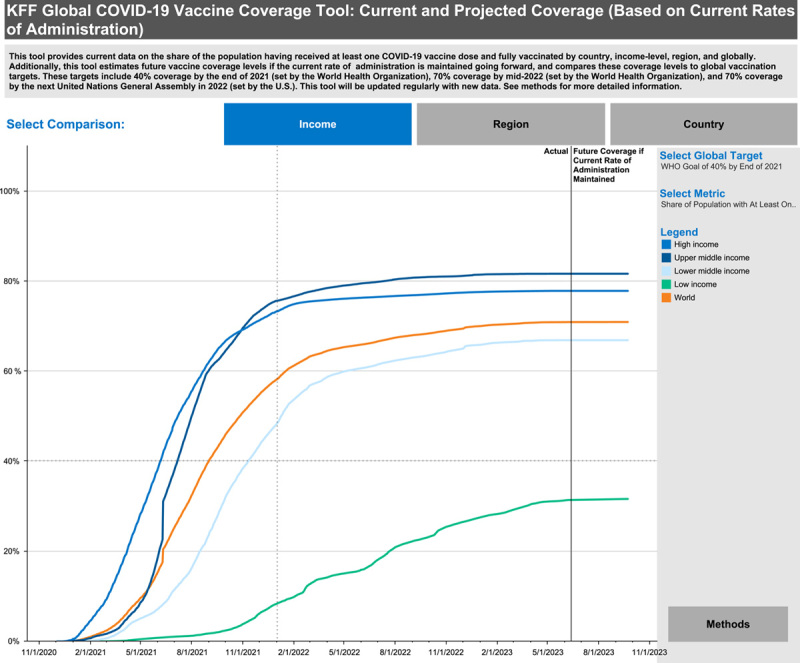
COVID-19 vaccine coverage based on income of countries. Obtained from^46^.

Countries with different levels of income vary in vaccine uptake (Fig. 2)^46^. In a 2020 study, middle-income countries showed a relatively high tendency toward acceptance, such as Brazil, India, and South Africa, as well as results showed that a lower household income is associated with a greater level of hesitancy in 11 countries, while a loss of income due to the pandemic is positively associated with vaccine acceptance in four countries^17,18^.

## COVID-19 vaccination hesitancy

So far, around 68.5% of the world’s population has received at least a single dose of the COVID-19 vaccine. In low-income countries, 24.6% of the population has received at least a single dose. Vaccines have been administered in 12.99 billion doses worldwide, with ~2.19 million doses administered each day. Around 5 billion people are fully vaccinated worldwide. Despite that, many people are hesitant to get vaccinated. Vaccine hesitancy, combined with public misinformation, may pose a significant problem^47^. In general, vaccine hesitancy is one of the top ten threats to global health in 2019, according to the WHO^48^.

Despite the availability of vaccines that effectively reduced the mortality rates of COVID, convincing people to receive the vaccine was extremely challenging^49^. According to the WHO, vaccine hesitancy is the ‘delay in acceptance of vaccines or refusal of vaccines despite the availability of vaccination services’^50^. This phenomenon showed different degrees according to region, sex, and other factors. In the USA, the overall vaccine acceptance rate ranged from 12 to 91.4%, showing an increase from 2020 to 2021^51^.

**Figure 3:**
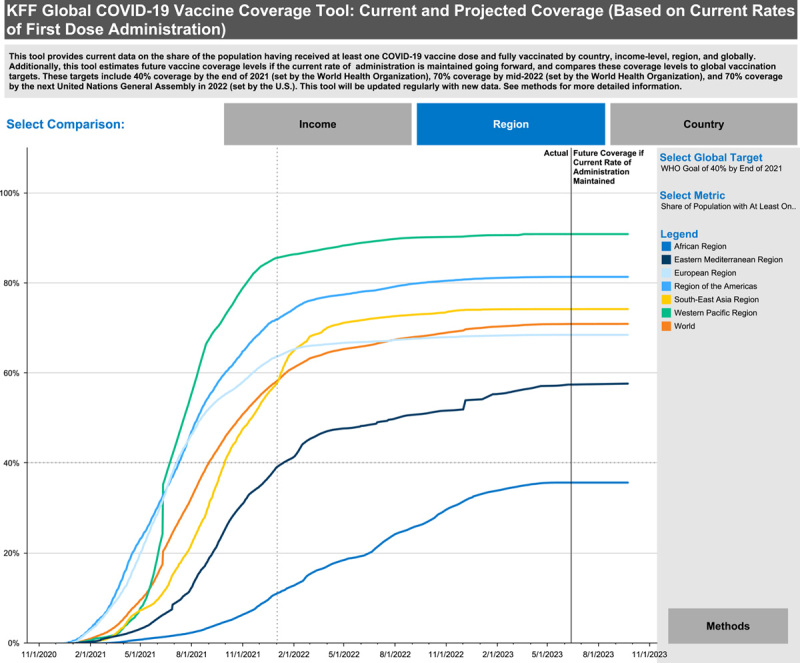
COVID-19 vaccine coverage among different regions worldwide. Obtained from^46^.

Vaccine uptake and hesitancy are considerably variable among different regions worldwide (Fig. 3)^46^. In Canada, only a minority of people were hesitant to get vaccinated; a recent study conducted in October 2022 found that 12% of 5007 participants were hesitant to get the COVID-19 vaccination^52^. In Africa, the vaccination acceptance rate ranged from 6.9 to 97.9%^53^. In Algeria, for instance, among 787 participants in a study conducted by Lounis *et al*.^54,^ 23.8% of respondents showed hesitancy in vaccine receiving even though the study was conducted during the third wave with a large number of cases and deaths. In South Asia, in a study conducted by Hawlader *et al*.^55^ that included 18 201 participants, almost 30% of participants were still hesitant about vaccine intake and its effectiveness. This concludes that overall and globally, almost a fourth of the population showed strong hesitancy about the COVID-19 vaccination^56^.

According to a recent study, overall vaccine hesitancy decreased from 28.5 to 24.8%, while in South Africa, the United States, Nigeria, and Russia, hesitancy increased compared to a year earlier survey. Notably, among those who stated that they were hesitant to vaccinate, potential vaccine acceptance was more likely to occur if recommended by one’s doctor than if recommended by the employer^18^.

### Associated burdens to COVID-19 vaccine hesitancy

Not only has the COVID-19 pandemic had a negative impact on the national and international economies, but COVID vaccination can also cause economic stress in either developed or developing countries. Any delay in vaccination could affect hundreds of thousands of people, negatively impacting the global economy^57^. On the other side, COVID-19 vaccination reduced healthcare costs by 60%, decreased consumption of hospital facilities^58,^ improved clinical outcomes^59,^ and decreased the number of deaths^60^.

### Determinants of COVID-19 vaccine hesitancy

The decision to accept, postpone, or refuse vaccination is influenced by several factors, including political, cultural, ecological, healthcare system, historical, and socioeconomic issues.

#### Sociodemographic determinants

**Figure 4:**
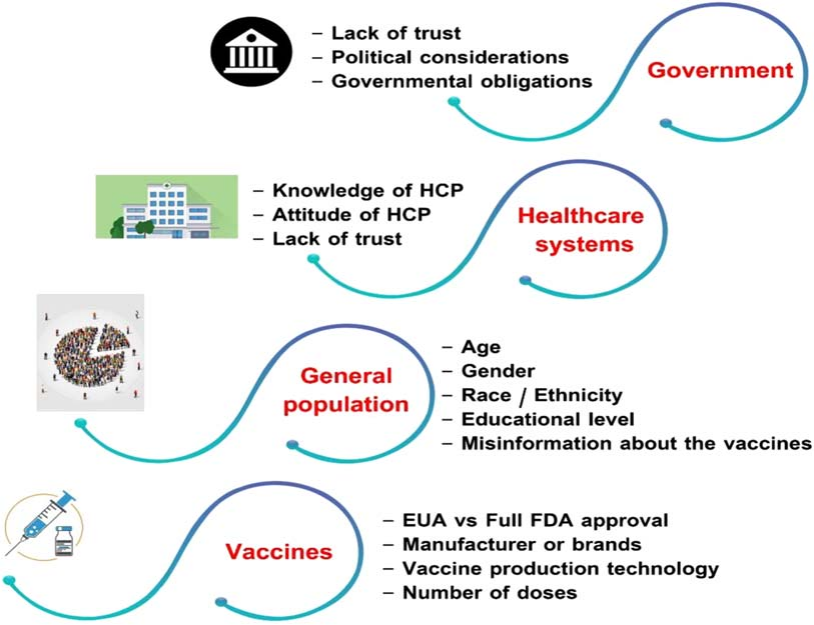
Factors that are related to vaccine hesitancy at the levels of vaccination process; government, healthcare system, population and the vaccine itself. HCP, healthcare provider; EUA, emergency use authorization; FDA, Food and Drug Administration.

Hesitancy shows distinct variation based on many factors (Fig. 4). Firstly, sex determinants: studies suggest that women have a higher vaccine hesitancy rate than men^56^. Secondly, age-related determinants: younger age groups showed stronger hesitancy than old people^61^. Older people were more likely to report that they would take a vaccine, whereas respondents 25–54 and 55–64 years of age were more likely to accept an employer’s vaccine recommendation^17^.

Thirdly, in terms of socioeconomic and education levels, studies have shown that poorly educated patients and people with lower socioeconomic conditions had higher COVID-19 vaccination hesitancy^62^. Vaccine acceptance was positively associated with higher levels of education. This finding is correlated with a lower percentage of vaccine hesitancy among HCPs globally compared to non-HCPs. Most recent studies do not investigate income as a potential influencer on vaccine acceptance^17,18^.

Furthermore, in a longitudinal study conducted in the UK in 2021 by Robertson *et al*., results reported that vaccine hesitancy was high in black (71.8%) and Pakistani/Bangladeshi (42.3%) ethnic groups. Even though the COVID-19 vaccine is more available in developed countries; people there were more hesitant as they had many doubts about its safety^53^. Minority race and ethnicity have affected people’s attitudes towards vaccination due to low trust in their government^63^. Regarding patients with comorbidities, individuals with serious comorbid conditions showed significant vaccine hesitancy^64^.

The high level of uncertainty among the majority of people can be justified by several causes^64^. These factors were different and often challenging to resolve, such as the mandatory nature of vaccines, the lack of trust in healthcare authorities and vaccine developers, perceived risks and fears, myths and confusing messages about vaccines and their effectiveness, and many others. These reasons for vaccination reluctance resulted in a significant delay in total community immunization, and thus the pandemic^65,66^.

#### Antivaccine attitude

Vaccine hesitancy is rooted in different levels of social, cultural, and historical contexts worldwide. The issue of low vaccine uptake by some groups is multifaceted. Fear of unknown future side effects is a major reason for antivaccine attitudes. Understanding their reasons for vaccine avoidance or hesitancy can aid in growing vaccination intentions among the hesitant population. It is necessary to discuss the causes and explanations for vaccine reluctance or hesitation. Confidence in the necessity of vaccinations has the strongest correlation with vaccine hesitancy^67^. The antivaccine attitude has a religious background for some people. For example, in the USA, religious conservatism, including evangelical and born-again Christianity, is associated with lower levels of trust in science, lower rates of vaccine uptake, lower vaccine knowledge, and higher levels of vaccine hesitancy^68^.

#### Vaccine-related determinants of vaccine hesitancy

The primary reasons for COVID-19 vaccination hesitancy include incomplete information, safety or efficacy worries, the place of origin of the vaccine producer, and the conviction that vaccine development and production should have been hastened. The public’s reluctance to receive the vaccine has also been observed to be influenced by several specific vaccine-related factors, including new vaccine introduction, administration method, schedule, cost, reliability, source of supply, knowledge base, and new recommendations for a current vaccine and the strength of these recommendations^9,69,70^.

In April 2020, the USA initiated operation warp to rapidly develop vaccines to counteract the spread of the virus and boost population immunity against the disease. The research finally led to the creation of vaccines in the USA by Pfizer-BioNtech, Moderna, and Johnson & Johnson. These three companies applied for emergency use authorization for their vaccines, and the FDA approved them^71^. The relatively rapid process with which COVID-19 vaccines were developed and authorized continues to raise concerns in addition to those regarding vaccine effectiveness, side effects, and safety, and whether any steps in vaccine development or regulation were bypassed^72^.

Providing convincing proof that a SARS-CoV-2 vaccine has been thoroughly evaluated, demonstrated to be efficacious, and has not been rushed into production is the most crucial step in ensuring vaccination uptake. One of the biggest obstacles to vaccination uptake is worries about commercial profiteering. Programs for developing and distributing vaccines that have reassuring names are more likely to win over the public’s trust^21^. Their level of knowledge and perception of the safety and efficacy of the vaccine was significantly associated with hesitancy^73^. Another study in the US revealed almost the same concerns or reasons for hesitancy^74^.

Full approval of COVID-19 vaccines by the FDA will encourage reluctant populations to be immunized^50^. Additionally, it was detected that confidence in the COVID-19 vaccine may alter depending on the vaccine type. The Pfizer-BioNTech vaccine received 39.6% of the preference and participants’ confidence, followed by the Oxford/AstraZeneca vaccine at 18.1% and the Sinopharm vaccine at 14.6%. Moreover, it was discovered that the greatest degree of confidence was in mRNA technology^75^. The multidose nature of the vaccination schedule was observed to increase vaccine hesitancy^76^.

#### Healthcare systems and vaccination hesitancy

French *et al*. set some guidelines and practices for governments and health systems to enhance the impact of a vaccination strategy, and to improve trust among the population, governments, and the healthcare system should act and put strategies in place to provide accurate knowledge about the COVID-19 vaccine. They included strategies for behavior change planning, audience targeting and segmentation, community engagement, marketing promotion strategies, vaccine access, and digital media strategy^77^.

On the other hand, Finney *et al*.^78^ provided evidence-based strategies to increase vaccine uptake and address vaccine hesitancy at the individual, interpersonal, and organizational levels. Interpersonal-level interventions address the interactions between clinicians and patients. Silver *et al*. found that higher levels of trust in one’s doctor were associated with significant and substantially greater odds of taking or seeking the COVID-19 vaccine. On the other side, lower levels of trust in the medical profession increased the odds of being a refuser compared to a taker^79^. Therefore, clinicians need to build trust with their patients, as they play an important role in their decisions.

HCPs level interventions; target members of the healthcare team and patients; as their role in the pandemic response, has grown because people who interact professionally or personally with HCPs who are vaccine-hesitant are likely to be less vaccine-compliant due to the low vaccination acceptance rates among HCPs. This is problematic because HCPs are the most dependable social resource for encouraging public immunization because they are in the best position to understand and address the worries and anxieties of hesitant patients as well as to explain the advantages of vaccination, especially during subsequent waves of the COVID-19 pandemic. This intervention includes training and educating clinicians as well as developing patient education materials (1503). HCPs must have adequate training and education regarding COVID-19 vaccination, including information about the vaccine’s safety, efficacy, and side effects. HCP must also be equipped with information regarding the need for the vaccine and its critical role in the strategic prevention of COVID-19 in individuals and among populations^78,80,81^

Organizational level interventions include the availability of standing orders for nurse visits, audit, and feedback interventions, which include regular feedback on the effectiveness of vaccination, reminder, and recall systems that contact patients directly to inform them if the vaccine is due, approaching due, or past due, point-of-care prompts, and home visits. This is along with dialog-based approaches such as social mobilization, trusted community representatives, and engagement with community leaders^82^.

Health officials must convince the public that vaccine development followed all established guidelines and that the process was not driven based on the reported maximum vaccine uptake. Public trust will be harmed, and vaccination acceptance will worsen, if folks say that health officials are pushing a vaccine into manufacturing.

Developing effective COVID-19 vaccination strategies requires a thorough study of the factors that influence vaccination decisions as they might differ significantly between people who accept and are determined to take the vaccine and those who do not. According to a recent global report, ~30% of those polled would refuse or are hesitant to take the vaccine if it becomes available. The COVID-19 vaccine’s acceptance rate varies across the globe and has been associated with some of these elements in numerous studies. Particularly among the region with the lowest rates of vaccination adoption is the Middle East^83^.

## Recommendation and further implications

### At the level of the target population

#### General population

Several strategies, such as increased sensitivity and awareness events, publishing data showing vaccine efficiency and its explanation to the general population, assuring rumors and false messages of denial, and generalizing vaccination in all possible structures such as schools, polyclinics, workplaces, and others^84–86,^ are useful in targeting population groups refusing or hesitating to take vaccines. In addition, differences based on sex, race, socioeconomic status, and other characteristics must be eliminated. No matter what social class someone is in, health officials should give everyone the same chances to get immunized^85,86^.

#### Special groups

Special groups such as women, are better informed and aware of immunizations. This is because women worry most about pregnancy and future pregnancy complications, which are believed to be caused by vaccine side effects^86,87^. Vaccination should consequently be provided to the entire juvenile, pediatric, and elderly population; vaccination of children, and the elderly is particularly crucial. After receiving the necessary health information, young people, who are more available and more motivated to get vaccinated, can be the focal point of several community projects. Therefore, adolescents who accept vaccines can play a vital role in influencing their parents, siblings, and younger children to acquire immunizations and educate them about their safety and efficacy^88^.

### At the level of healthcare systems

Increasing knowledge of COVID vaccine advantages, mechanisms, and adverse effects among HCPs will have positive results in the acceptance of vaccination and the spreading of vaccination among the population. So, publishing and spreading updated guidelines according to evidence-based medicine and specializing in easily accessible online sessions for discussion among healthcare workers help spread the appropriate knowledge of vaccines among HCPs^89^. Moreover, sharing data and statistics between healthcare systems over the world helps them build an excellent view^90^.

The rapid process of vaccine development raised many doubts about its efficacy and, more importantly, its safety among the population. The availability of vaccines and understanding of their importance were the main factors in gaining the trust of the population^91^. Vaccine education assists in educating hesitant groups about the importance of vaccination^92^. Misinformation on social media has had negative feedback against vaccination, so sharing true information through it will help to reach a large number of people all day^31,93^. If there is contact between people and healthcare systems, this will help gain any information about vaccination, remove their doubts, and increase the number of vaccinated people.

Overall, health authorities as well as the healthcare system should implement key interventions for hesitant groups to deliver accurate knowledge about vaccines and build trust with the community. When health systems and public health authorities recognize the public’s concerns and consider trust issues for vaccination, they can take action and make interventions for this issue^94^. By taking these approaches, the healthcare system will help people make decisions related to their vaccination according to their best protection and those of their families, based on evidence, trust, and health literacy.

### At the level of policies and governments

As the communities differ in their beliefs, values, and attitudes, they require different methods of intervention. Segmentation (division into groups that share the same beliefs and attitudes according to their sociodemographic characteristics) is an essential step. This can help authorities deal with each group with specific interventions that fit them^77^.

To get more people to accept and use vaccines and, as a result, reach high vaccination coverage, it is important to gain the public’s trust. Trust issues are important in people’s decisions about vaccination. It’s important to target populations that are more likely to have governmental mistrust, like ethnicities of a low minority, with specific communication efforts and specific programs. Participation in vaccination programs with local organizations, cultural and religious leaders, and accepted voices builds population trust^36,77,95^.

Public health authorities should design programs based on not only expert opinion but also fear, values, and the population’s perspective. These programs should be targeted and focused on the progression of the disease and the importance of vaccination. Besides, they should report accurate information transparently, especially regarding side effects, complications, and the safety of the vaccine^77,96^. Trials to reduce population hesitancy, on the other hand, should be done carefully and cautiously, as they may induce conspiracy beliefs among the population and cause people to become more attached to incorrect and useless information^36,77^.

### Future implications

We hope the world will not have to face such a disaster as the COVID-19 pandemic. However, we should stand tight and be prepared for any upcoming health issues. The COVID-19 pandemic offers a chance to examine the connection between the research system and its effects in both normal and emergencies. Different local context-related research demands may exist in different countries. Therefore, every nation’s research system should recognize these demands and real research priorities to generate data that can aid in managing and controlling the current crisis and preventing future ones.

To avoid spreading misleading information, the essential operations of the research system must perform more quickly and efficiently, hence the importance of active monitoring by quality control and assurance. For the upcoming challenges, it would be helpful to set priorities for budgeting based on urgent, short-term, and long-term needs, and to set up an observatory group to keep an eye on ‘already answered questions’ and ‘ongoing research’. This would help manage research priorities well and keep projects from being repeated^97–99^.

Future research must focus on the infrastructure upgrades that must be made, the training of human resources, and the generation or updating of relevant evidence. A lesson learned is to conduct context-specific behavioral and social research to understand perceptions and causes of vaccine hesitancy better. That will make it possible to create future programs that are effective at building public trust in vaccines and immunization. This will help boost vaccination rates and keep the public and investigators from getting confused in the future.

## Conclusions

The COVID-19 pandemic has been a rough challenge for all nations at many levels. It started in a single city but spread to almost the entire world. Millions and millions suffered from its effects and complications; others died. Urgent interventions were made to interfere with the rapid dissemination of respiratory disease with vaccination being the highest priority. Some of these interventions have been effective, others have not, and others have been improved. We must record these steps and work on their improvement so that we do not have to suffer the serious morbidity and mortality rates of such diseases. This must be done very carefully and cautiously concerning each system’s qualifications and requirements. Governments must consider the population’s perceptions and need to have the best outcome quickly.

## Ethical approval

Ethical approval was not required for this review.

## Informed consent statement

Not applicable.

## Sources of funding

This research received no external funding.

## Author contribution

H.T.A., Y.A., M.A.R., M.B., T.A.R., M.M.S., M.L., Z.A.S., A.A. (Ahmed Abdullah), and A.A. (Abdelaziz Abdelaal): conceptualization and writing-the original draft; H.T.A. and A.A. (Abdulrahman Allahham): writing-designing the figures; A.A. (Abdelaziz Abdelaal), S.A.A., J.J.P., A.M., and R.S.: proofing and revision. All authors have read and agreed to the published version of the manuscript.

## Conflicts of interest disclosure

The authors declare no conflict of interest.

## Research registration unique identifying number (UIN)

1. Name of the registry: Not applicable.
2. Unique Identifying number or registration ID: Not applicable.
3. Hyperlink to your specific registration (must be publicly accessible and will be checked): Not applicable.

## Guarantor

Ranjit Sah.

## Data availability statement

Not applicable.

## Provenance and peer review

Not commissioned, externally peer-reviewed.

## Institutional review board statement

Not applicable.
